# Changes in Physicians' Perceptions and Practices on Neonatal Pain Management Over the Past 20 Years. A Survey Conducted at Two Time-Points

**DOI:** 10.3389/fped.2021.667806

**Published:** 2021-06-04

**Authors:** Eleni Agakidou, Konstantia Tsoni, Theodora Stathopoulou, Agathi Thomaidou, Maria Farini, Angeliki Kontou, Paraskevi Karagianni, Kosmas Sarafidis

**Affiliations:** 1st Department of Neonatology and Neonatal Intensive Care, School of Medicine, Aristotle University of Thessaloniki, Ippokrateion General Hospital, Thessaloniki, Greece

**Keywords:** preterm neonates, non-pharmacological interventions, neonatal pain, pain assessment tools, mechanical ventilation, procedural pain, analgesics, sedatives

## Abstract

Intense research for more than three decades expelled the view that neonates do not experience pain. The aim of this survey was to investigate whether the Greek physicians involved in neonatal intensive care have changed their perceptions regarding neonatal pain, adapting their management practices to the knowledge that have emerged in the past 20-years. This study is a survey conducted at two time-points, 20 years apart. Anonymous questionnaires were distributed to 117 and 145 physicians working in neonatal intensive care units (NICUs) all over Greece in years 2000 and 2019, respectively. The response rate was 90.6 and 80.7% in 2000 and 2019, respectively. All respondents, at both time-points, believed that neonates experience pain, which has serious acute and long-term consequences, while the vast majority considered analgesia-sedation (A-S) during painful interventions as obligatory. Utilization of NICU protocols and pain assessment tools remained low although increased significantly between 2000 and 2019. The use of systemic A-S postoperatively was high at both time-points, while its implementation in infants subjected to prolonged pain, specifically mechanical ventilation, increased significantly by 2019. Systemic or local analgesia for acute procedural pain was used by lower proportions of physicians in 2019, except for the tracheal intubation. In contrast, the use of sweet solutions and non-pharmacological measures prior to or during bedside procedures significantly increased over time. Opioid administration significantly increased, while a shift from morphine to fentanyl was observed. International literature and perinatal–neonatal congresses were stated as the main sources of updating physicians' knowledge and improving management practice on neonatal pain prevention and treatment. In conclusion, Greek NICU-physicians' perceptions that neonates can experience pain with potentially serious acute and long-term consequences remained strong over the past 20 years. Although physicians' practices on neonatal pain management improved, they are still suboptimal, while significant differences exist among centers. Continuing education, globally accepted management protocols, and readily applied pain assessment tools would further improve the management of procedural pain and stress in neonates.

## Introduction

Neonates receiving intensive care are exposed to numerous painful or stressful procedures (1–3). However, misconceptions of the past on neonatal pain perception erroneously influenced clinical management for years. During the 80's and early 90's, the prevailing belief was that neonates do not feel pain due to the immaturity of their nervous system, despite the fact that, as later demonstrated, the fetus can feel pain even in the second trimester of gestation, while neonates are capable of mounting cardiorespiratory, hormonal, and metabolic stress responses similar or even more intense than adults (4–7). Moreover, preterm infants are subjected to prolonged periods of hyper-responsiveness even to non-painful stimuli due to reduced pain threshold and increased sensitivity of the posterior horns of the spinal cord (8). Cumulative evidence also suggests that prolonged exposure to painful events in the neonatal period is associated with significant acute and long-term consequences (9, 10). Ranger and Grunau in their review article reported that exposure of preterm infants to repeated or chronic pain and/or stress adversely affects brain development and activity, independently of other detrimental factors associated with prematurity. Specifically, they reported that repeated pain/stress had been associated with altered cognitive and motor development, increased incidence of internalized behaviors (depressive and/or anxious), and changes in stress hormone expression (9). More recently, Cong et al. examined the relationship between the exposure of preterm infants with a gestational age from 28 to 32 weeks to painful/stressful procedures during the first 28 days of life with their neurobehavioral functioning at 34–48 weeks post-conceptionally, using the Neonatal Intensive Care Unit Network Neurobehavioral Scale (NNNS) instrument. They found that both acute and chronic pain/stress was significantly associated with neurobehavioral outcomes after adjustment for illness severity and other co-factors (10).

Fortunately, international scientific societies realized early enough the importance of preventing and managing pain and stress in neonates providing relevant guidelines (2, 11–14). In this context, a lot of effort has been made during the last decades through international and national neonatal/perinatal congresses and published articles to educate health care professionals in the field of Neonatology on the consequences and management of neonatal pain. Despite that, there are still marked differences among Neonatal Intensive Care Unit (NICU) practices and health professionals' views as regards the administration of analgesia and sedation (A-S) in neonates worldwide (2, 12, 13, 15, 16).

The aim of this survey was to investigate whether the Greek physicians involved in neonatal intensive care (NICU-physicians) have changed their perceptions regarding the neonatal procedural pain adapting their management practices to the data that have emerged in the past 20-year period. To this aim, we conducted a national survey with anonymous questionnaires distributed among NICU-physicians throughout Greece in the years 2000 and 2019.

## Materials and Methods

### Study Design

This survey is a national, observational, cross-sectional study conducted at two time-points, 20 years apart (2000 and 2019).

### Study Population

Questionnaires were sent to the directors of 13 and 15 level III NICUs of Greece in 2000 and 2019, respectively. The survey addressed only the medical staff, namely neonatal specialist doctors (certified neonatologists), pediatricians attending a fellowship program in Neonatology (trainee neonatologists), and pediatricians working in NICUs but without national or international accreditation in Neonatology beyond common trunk pediatric training.

### Methodology

The self-administered, multiple choice questionnaire contained four domains; demographic characteristics (four items); physicians' personal opinions regarding the neonates' ability to experience pain and stress and the necessity for pain prevention and treatment (four items); their practices in the NICU setting for the evaluation and management of pain and stress postoperatively and during painful procedures (20 items); and their suggestions regarding the improvement of neonatologists' practice on managing neonatal pain (three items) (Supplementary Table 1). Questions related to the sources of relevant information in the intervening 20 years were added to the 2019-questionnaire. The questionnaires' design was based on published data and was reviewed by experienced neonatologists for completeness or potentially ambiguous questions. The reviewers' suggestions were taken into consideration for the construction of the final version. The questionnaires were sent by mail and e-mail in years 2000 and 2019, respectively, following a personal communication with the NICU directors who were tasked with the distribution of the questionnaires to the medical staff and collection of the completed ones. Attached to the questionnaires, there was also an explanatory–consent letter in which the aims of the survey and the way to fill-in the questions were described.

All work was conducted in accordance with the declaration of Helsinki of 1975 [https://www.wma.net/what-we-do/medical-ethics/declaration-of-helsinki/], revised in 2013. The Ethics Committee of our Institution waived the need for approval as the questionnaire used was anonymous and the respondents could not be identified from the limited demographic data included, while the vast majority of the study population did not belong to the medical staff of our institution.

### Data Analysis

Continuous data were expressed as medians and interquartile range and categorical data as counts and proportions. Comparisons were performed using the Mann-Whitney test and Fisher's Exact test, as appropriate. Multiple regression analysis models were constructed to investigate the association of the main outcome measures with the time-point of the survey after controlling for potential co-factors. Separate models were designed with dependent variables the physicians' practice in each painful/stressful procedure and the kind of intervention and medications. The time-point, the participant's sex, and professional experience (working years in the NICU setting) were entered into all regression models as independent factors. Analyses were performed using the Generalized Linear Models—binary or ordinal logistic response, based on whether the dependent variables were categorical or multi-class ordered variables, i.e., categorical variables following an order (never, often, always). The General Linear Model—multivariate was used for analyses including multiple dependent variables, such as non-pharmacological interventions. The limit of significance was set at *p* = 0.05. The IBM SPSS software (version 23) was used for data analysis.

## Results

### Demographic Data

Questionnaires were distributed to 117 and 145 physicians in 2000 and 2019, respectively, while the response rate was 90.6 and 80.2%, respectively. A considerable proportion of the respondents changed between the years 2000 and 2019 as most of those who participated in the 2000 survey had left active service by 2019. We cannot know the exact proportion of the study population that changed, because of the anonymity of respondents. Nevertheless, the sex distribution, expertise level (neonatologists, pediatricians working in NICU, and trainees) and working years in a NICU did not differ significantly between the two time-points (Table 1). The use of standard NICU protocols for the management of procedural pain and stress increased over time (*p* < 0.001) while pain assessment tools were used only in 2019 by 28.7% of the respondents (Table 1). The most commonly utilized tool was the NIPS (Neonatal Infant Pain Scale) which was used by 20 of the 33 physicians utilizing pain assessment scales (60.6%). Additionally, five respondents used the PIPP (Premature Infant Pain Profile), five the CRIES (Cry, Requires oxygen, Increased vital signs, Expressions, Sleeplessness), four the NFCF (Neonatal Facial Coding System), two the EDIN (Échelle Infant Douleour Nouveau-Ne), one the COMFORT, and one respondent used the N-PAS (Neonatal Pain Agitation & Sedation Scale). Pain assessment was performed exclusively by the physicians.

**Table 1:** Demographic data and perceptions of the respondents.

	**Year of survey**		***p*^**a**^**	**OR**	**95% CI**	
	**2000**	**2019**			**Lower**	**Upper**
Respondents (*n*)	106	117				
Public/private NICU	91 (85.8)	100 (85.5)	1.0	0.970	0.458	2.053
Male sex	34 (32.1)	31 (26.5)	0.38	0.763	0.428	1.362
Level of experience			0.92	N.A.		
*Neonatologists*	77 (72.6)	88 (75.2)				
*Pediatricians*	9 (8.5)	7 (6.0)				
*Fellows*	20 (18.9)	22 (18.8)				
Years of working in NICU (median [IQR])	13.5 (15)	9 (14)	0.64	N.A.		
Perceptions						
*Neonates feel pain/stress*	106 (100)	117 (100)	1.0	N.A.		
*Pain/stress may have adverse effects*	103 (98.1)	117 (100)	0.22	N.A.		
*Neonatal pain should be treated*	98 (90.5)	116 (99.1)	0.015	9.469	1.164	77.034
A-S postoperatively			0.007	N.A.		
*Not needed*	6 (5.7)	1 (0.9)				
*Important*	35 (33)	26 (22.2)				
*Obligatory*	65 (61.3)	90 (76.9)				
Use of protocols	30 (28.3)	62 (53.0)	<0.001	2.856	1.636	4.985
Use of pain assessment tools	–	33 (28.7)	N.A.			

*Values are expressed as counts (%) unless otherwise stated;*

a *Fisher's exact test or Mann—Whitney test; A-S, analgesia- sedation; CI, confidence interval; IQR, interquartile range; N.A., non-applicable; NICU, neonatal intensive care unit; OR, odds ratio*.

### Physicians' Perceptions

All physicians believed that neonates are capable of experiencing pain and stress, which may have significant acute and long–term effects if untreated. Significantly higher proportions of the 2019-respondents stated that neonatal pain should be efficiently treated (*p* = 0.015) and considered postoperative analgesia as important or obligatory (*p* < 0.001, Table 1).

### Postoperative Pain Management

The vast majority of respondents at both time-points stated that they administered A-S following major surgeries (96.2 and 99.1 % in 2000 and 2019, respectively) without any significant difference between the two time-points (Table 2). The opioids were the most commonly used medications at both time-points (84.0 vs. 82.9 %, *p* = 0.859). The frequency of morphine utilization significantly decreased while the frequency of fentanyl increased from 2000 to 2019 (*p* < 0.001 and *p* = 0.015, for morphine and fentanyl, respectively). The use of paracetamol significantly increased from 45.3 to 62.5%, between the two time-points (*p* = 0.001, Table 3).

**Table 2:** Physicians' practices for management of postoperative and procedural pain/stress.

	**Year of survey**		***p*^**a**^**	**OR**	**95% CI**	
	**2000 (*n* = 106)**	**2019 (*n* = 117)**			**Lower**	**Upper**
A-S postoperatively	102 (96.2)	116 (99.1)	0.19	4.55	0.500	41.36
A-S during MV	88 (83.0)	113 (96.6)	0.001	5.778	1.888	17.686
A-S combinations during MV						
*Analgesics only*	33 (31.1)	38 (32.5)				
*Sedatives only*	18 (17.0)	5 (4.3)	<0.001	N.A.		
*Analgesics + sedatives*	37 (34.9)	70 (59.8)				
*None*	18 (17.0)	4 (3.4)				
Analgesics during chest drainage	76 (71.7)	91 (79.1)	0.21	0.668	0.360	1.238
Systemic/local A-S for acute procedural pain	91 (88.3)	87 (78.4)	0.07	0.478	0.225	1.015
Local anesthesia	83 (78.3)	23 (19.8)	<0.001	0.069	0.036	0.131
Sweet solutions orally	32 (30.2)	88 (75.2)	<0.001	7.017	3.890	12.659

*Values are expressed as counts (%) of positive responses;*

a *Fisher's exact test; A-S, analgesia-sedation; CI, confidence intervals; MV, mechanical ventilation; N.A., non-applicable; OR, odds ratio*.

**Table 3:** Number (percentage) of physicians using certain analgesics and sedatives postoperatively and during specific bedside procedures.

	**Postoperative pain**			**Prolonged pain**						**Acute pain**		
				**Mechanical ventilation**			**Chest drainage**			**Non-emergent tracheal intubation**		
	**2000**	**2019**	***p*^**a**^**	**2000**	**2019**	***p*^**a**^**	**2000**	**2019**	***p*^**a**^**	**2000**	**2019**	***p*^**a**^**
*N*	106	117		106	117		106	96		106	117	
Opioids	89 (84.0)	97 (82.9)	<0.001	71 (67.0)	106 (90.6)	<0.001	17 (16.0)	69 (71.1)	<0.001	42 (39.6)	45 (38.5)	0.89
*Morphine*	50 (47.2)	27 (23.1)	0.31	28 (26.4)	22 (18.8)	0.20	3 (2.8)	6 (6.3)	0.31	19 (17.9)	6 (5.1)	0.003
*Fentanyl*	59 (55.7)	87 (74.4)	<0.001	45 (42.5)	101 (86.3)	<0.001	14 (13.2)	65 (67.0)	<0.001	24 (22.6)	41 (35.0)	0.055
Paracetamol	48 (45.3)	79 (67.5)	<0.001	–	–	–	26 (24.5)	6 (6.3)	<0.001	–	–	–
Sedation	–	–	0.67	51 (48.1)	69 (59.0)	0.11	2 (1.9)	3 (3.1)	0.67	32 (30.2)	22 (18.8)	0.060
*Midazolam*	–	–	–	27 (25.5)	63 (53.8)	<0.001				20 (18.9)	9 (7.7)	0.016
*Diazepam*	–	–	–	14 (13.2)	7 (6.0)	0.07				12 (11.3)	6 (5.0)	0.14
*Phenobarbital*	–	–	–	2 (1.9)	11 (9.4)	0.021				0	7 (6.0)	0.015

a *Fisher's exact test*.

### Management of Prolonged Procedural Pain

Mechanical ventilation is the main procedure causing prolonged pain in sick neonates. A significantly higher proportion of the 2019-respondents used A-S during mechanical ventilation (83.0 vs. 96.6 %, *p* = 0.001, Table 2). Analysis of medications used during mechanical ventilation revealed significant changes between 2000 and 2019. The use of opioids increased significantly (*p* < 0.001) due to significant increase of fentanyl administration (*p* < 0.001). The frequency of sedation during mechanical ventilation did not change significantly, but higher proportions of 2019-respondents used midazolam and phenobarbital compared to the 2000-respondents (*p* < 0.001 and *p* = 0.021, for midazolam and phenobarbital, respectively). Muscle relaxants were more frequently used in 2019 (29.1%) than in 2000 (17.0%, *p* = 0.032). Nevertheless, further analysis revealed that only 7.5 and 4.3% of the respondents used muscle relaxants frequently, while 9.4 and 24.8% of them only rarely, respectively in the years 2000 and 2019.

Chest drainage consists another cause of prolonged pain in sick neonates (14). The proportion of participants using A-S to infants during chest drainage was high at both time-points (72 and 79 %, in the years 2000 and 2019, respectively, Table 2). Opioids were the most commonly used analgesics, while sedation was administered very rarely (1.9 and 3.1 % in time-points 1 and 2, respectively) (Table 3).

### Management of Acute Procedural Pain

**Figure 1:**
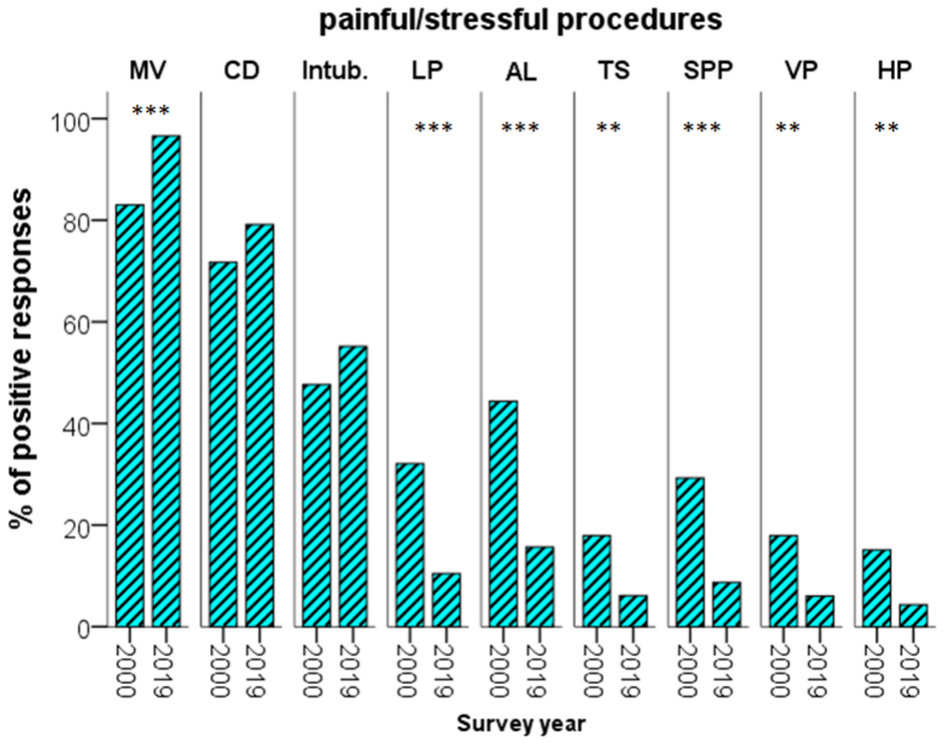
Percentage of the respondents using analgesia—sedation prior to or during painful/stressful procedures, at the two time-points. AL, arterial line; CD, chest drainage; HP, heel prick; Intub., intubation; LP, lumbar puncture; MV, mechanical ventilation; SPP, suprapubic paracentesis; TS, tracheal suction; VP, venous puncture; **p < 0.01; ***p < 0.001.

The use of systemic A-S and/or local anesthesia prior to or during procedures causing acute pain or stress decreased significantly over time, while remained unchanged for non-emergent intubation (Figure 1). The proportion of respondents applying local anesthesia to alleviate acute procedural pain considerably decreased, while the use of sweet solutions increased by 2019 (Table 2). There were significant changes as for the medication used prior to non-emergent tracheal intubation (premedication). The use of opioids did not change significantly, albeit a shift from morphine to fentanyl was observed. The frequency of sedation prior to non-emergent intubation tended lower by 2019, especially regarding midazolam administration (Table 3).

### Non-pharmacological Interventions

**Figure 2:**
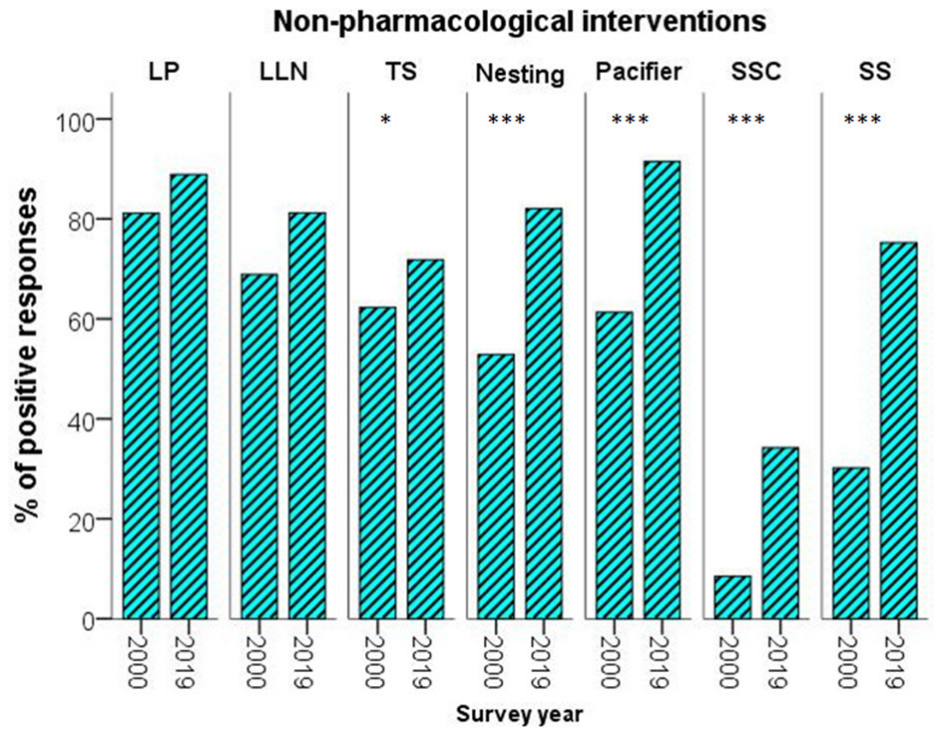
Percentage of the respondents using non–pharmacological measures at the two time-points. LLN, low light & noise; LP, less procedures; SS, sweet solutions; SSC, skin-to-skin care; TS, tactile stimulations; *p < 0.05; ***p < 0.001.

Implementation of non-pharmacological modalities during minor procedures increased significantly by 2019. Utilization of low light and noise in the NICU, nesting, pacifiers, skin-to-skin care, and gentle touching increased significantly by 2019, while a comparable, high proportion of the respondents tried to reduce the number of procedures and applied tactile stimulation at both time-points (Figure 2).

### Multiple Regression Analyses

Multiple regression analyses confirmed the significant independent association of the time-point with NICU-physicians' views and practice on neonatal pain management found on bivariate analysis (Supplementary Tables 2, 3).

### Sources of Knowledge and Physicians' Comments

Articles published in international literature (84%), lectures at international (59%) and national congresses (38%), intra-departmental lectures (46%), and discussions during ward rounds (38%) were stated as the main sources of updating knowledge on neonatal pain prevention and management. In their comments, almost all physicians highlighted the need for more educational programs in order to update their knowledge and requested inclusion of sessions related to neonatal pain in all relevant, large scale neonatal/perinatal congresses. Lastly, the most frequent request concerned the development of national protocols on pain assessment and management by specific scientific committees and expert working groups as well as their implementation in all NICUs.

## Discussion

This is the first national survey to examine whether the accumulated knowledge during the last two decades on neonatal pain and stress has affected NICU-physicians' opinions and practices concerning their management in sick neonates. The vast majority of physicians working in NICUs strongly believe that neonates are capable of perceiving pain, which may have short- and long-term consequences, a perception that has remained unchanged over the last 20 years. We also observed significant changes in physicians' practices mainly with respect to the use of A-S prior to or during painful or stressful procedures and application of non-pharmacological interventions. Although below the suggested standards, an improvement in the utilization of pain scales was observed over time.

Our findings are in line with previous surveys in other countries showing that the great majority of health professionals involved in neonatal care do believe that preterm infants experience pain and stress, which should be effectively prevented and treated (16, 17). However, management of neonatal pain remains suboptimal probably due to the lack of fixed protocols, as indicated by the responses of about 50% of the NICU-physicians. The lack of generally accepted protocols for preventing and treating neonatal pain may have contributed to the great variability in the management of neonatal pain and stress among and within countries (18, 19).

Reliable pain assessment could contribute to a more reasonable and effective use of A-S. Although several pain-assessment tools have been evaluated in neonates (6, 8, 11, 20–23), there are inherent difficulties in assessing neonatal pain, especially in the most preterm infants, largely associated with their physical and behavioral immaturity, while environmental factors (noise and temperature), other states (agitation, sleep, hunger, prior exposure to painful stimuli), and the examiners' experience may all affect the accuracy of the tools (8, 14, 21, 22, 24). Previous studies involving different countries showed that a variable percentage of NICUs, ranging between 6 and 87 %, use pain assessment tools (21, 22, 24, 25). According to our findings, pain assessment tools were not used in 2000, while were used by < ⅓ of physicians in 2019. Presumably, pain management without the use of pain scales was either applied according to fixed NICU protocols or was largely at the discretion of the physicians, who decided according to the clinical status and the possible source of pain/stress. Shortage in medical and nursing staff was and still remains the most important reason that, in our view, hinders the application of neonatal pain scales on a regular basis during the everyday care in Greece. The fact that pain assessment was performed exclusively by the medical personnel provides further evidence to the latter assumption.

### Pharmacological Management

#### Management of Postoperative Pain

More than three decades ago, Anand et al. documented an increased endocrine response to major surgeries, which could be attenuated by deep anesthesia and postoperative analgesia in neonates (4). Currently, it is generally accepted that postoperative pain must be effectively treated and that all NICUs should implement pharmacological approaches to this end (11, 24). In our study, although the vast majority of physicians at both time-points use analgesics for postoperative pain, it is still disappointing that a small, but not negligible, proportion of physicians (5.6%) does not consider postoperative analgesia as mandatory.

#### Management of Prolonged Pain

The main procedures causing prolonged pain in sick neonates are the mechanical ventilation and the chest drainage (14). Mechanical ventilation causes stress, or even pain, in neonates leading to asynchrony with the ventilator, incidents of hypoxia, air-leak syndromes, and arterial pressure fluctuations. Administration of A-S to neonates on invasive mechanical ventilation has been shown to improve ventilator synchrony, while findings on the effectiveness on pain relief and other clinical outcomes are controversial (18, 24). The percentage of NICUs administering A-S to ventilated neonates ranges between 67% and 100% worldwide (18, 26). In the EUROPAIN study, the mean frequency of S-A during invasive ventilation was 81.5% with a wide variation among the European centers (18). In our study, the proportion of physicians administering A-S to ventilated neonates increased significantly during the last 20 years, despite the concerns as for the adverse effects and the limited data supporting a beneficial effect of A-S (27). In line with our results, a retrospective cohort study by Zimmerman et al. on the use of A-S in ventilated infants over a 15-year period (1997–2012) showed a progressive increase in opioid and sedative use (28). Worth noting that the European consensus guidelines for the management of respiratory stress syndrome in neonates do not recommend the routine use of A-S during mechanical ventilation (29).

In the present study, a considerable proportion of respondents administered analgesics during chest drainage at both time-points, while sedation was given very rarely. These results, however, should be cautiously interpreted given the high frequency of S-A administration in infants receiving mechanical ventilation. Therefore, it is possible that neonates are already given analgesics by the time of the pneumothorax development.

#### Management of Acute Procedural Pain

Sick neonates frequently undergo tracheal intubation, which causes extreme pain and stress. The American Academy of Pediatrics recommended premedication, including analgesics, atropine, and muscle relaxants, for neonates subjected to elective tracheal intubation (30). Despite the existing recommendations, previous studies reported that the frequency of premedication prior to elective intubation ranges widely (26, 30–33). An online survey among neonatologists concerning premedication for elective tracheal intubation showed that 72% of the respondents considered premedication as obligatory, while only 34% of them used it frequently (33). In our study, only half of the physicians used A-S prior to non-emergent intubation without any significant difference between the two time-points, with opioids being the most frequently used analgesics.

Regarding management acute pain caused by other bedside procedures, our results showed that the use of A-S was significantly lower in 2019 than in 2000. This was also the case for local anesthesia prior to venopuncture and heel prick, despite the reported data showing that minor procedures induce pain responses (34). The decrease in utilization of systemic and local analgesia and anesthesia to alleviate acute procedural pain could be partly attributed to the increased administration of systemic analgesia and sedation to ventilated neonates as well as the increased implementation of non-pharmacological measures.

#### Changes in Medications Used

The opioids morphine and fentanyl were the most frequently employed analgesic agents in neonates (1, 19), and so they were in our study at both time-points. Morphine has been shown to reduce stress responses but its analgesic effectiveness on preterm neonates remains controversial (19, 27). As a matter of fact, results of the Procedural Pain in Premature Infants (POPPI) study showed no beneficial effect of morphine on procedural pain in preterm neonates, whereas a higher number of morphine treated neonates required non-invasive ventilation due to apneas compared to the placebo group (27). Any hesitation to administer opioids may be partly attributed to concerns regarding the potential acute side effects but also the rather prevailing skepticism on how they might affect the developing nervous system of preterm neonates in particular. However, the potential adverse effects of A-S should be weighed against the harmful effects of pain on the developing nervous system (9, 10, 24). Possibly, exposure to low doses of opioids might be safe as regards the long-term neurodevelopment (24).

In the context of the existing controversy on the effectiveness and safety of opioids, alternative analgesics, mainly paracetamol (acetaminophen), have been used for treatment of postoperative and procedural pain in neonates. The safety profile of paracetamol might have contributed to its increasing use in term and preterm neonates, despite the off-label use in this population (35). The rising use of paracetamol as adjunct to opioids following major surgeries between the two time-points of our study also supports the findings of other relevant publications. A recent meta-analysis and a review showed that existing data are not sufficient to support a role of paracetamol in reducing the procedural pain in neonates but may reduce the need for morphine following major surgery (36, 37).

Sedatives are often used as adjunct to analgesics and rarely alone in minor procedures, as they exert no analgesic action (2). Midazolam is a short-term acting benzodiazepine, which replaced diazepam in the NICUs due to its pharmacological advantages and the absence of active metabolites (2, 12). The current survey showed that midazolam was the most abundant sedative utilized by the NICU physicians, with its use increasing over time, despite the existing data against its safety (38).

### Non-pharmacological Approaches and Oral Sweet Solutions

The reported non-pharmacological management of procedural pain ranges between zero and 89% (1, 2, 25, 39, 40). The proportion of the respondents utilizing non-pharmacological measures in the present survey increased significantly between 2000 and 2019 from 84 to 97%. At both time-points, the great majority of the respondents stated that they minimized the number of bedside painful procedures, while a high proportion of them used more than one non-pharmacological measures. Environmental strategies such as reduction of the light and noise intensity were employed by a higher percentage of physicians in 2019. A promising finding was also the significant increase in the use of certain behavioral strategies (nesting, non-nutritive sucking, skin-to-skin care, and gentle touching) over the 20-year period of the study. The skin-to-skin care has been proposed as a non-pharmacological approach to alleviate neonatal pain, induced mainly following heel trick and venipuncture. As concluded in a Cochrane meta-analysis, skin-to-skin care may potentially have a beneficial effect on procedural pain, despite the controversial results of the studies (41). The general belief, nonetheless, is that the specific intervention can cause no harm to the baby. The percentage of physicians applying this measure in the present study, although has significantly increased over the years (8.5 vs. 34%, respectively), remained not only low, but skin-to-skin care was actually found to be the least frequently applied among the non-pharmacological approaches. Lack of available facilities and extra space in the NICU setting dedicated to the parents and, in general, restrictions in the hospital infrastructures and design could provide an explanation for this finding which is rather disappointing. A recent review concluded that there is a variety of culturally—based non-pharmacological measures with potential benefits in releasing procedural pain, which however need further, standardized investigation (42). Non-pharmacological interventions are believed to exert their analgesic effect by blocking nociceptive transduction/transmission or via the activation of descending inhibitory pathways (6). However, varying effectiveness has been reported. Existing evidence suggests that the combination of non-pharmacological measures may be more effective (2, 43, 44).

The use of oral sweet solutions (sucrose or glucose) deserves further notice. We observed a significant increase in their use for procedural pain over time from 35 to 78%. Sweet solutions have been proved to be effective in reducing pain during minor procedures, especially when combined with non-pharmacological measures (45). However, as they are actually medications and should not viewed as non-pharmacological measures, there are important, unresolved issues as regards the mechanism of action, efficacy, optimal analgesic dose for different procedures, and long-term complications (46).

### Sources of Knowledge Update and Physicians' Suggestions for Further Improving Neonatal Pain Management

The last part of the questionnaire recorded the sources used by the NICU-physicians for updating and expanding their knowledge on neonatal pain and their suggestions for more effective pain/stress prevention and treatment. Obviously, international publications and congresses play a key role in understanding the importance of effective management, while educational meetings within the NICU setting, ward rounds, and the national neonatal/perinatal congresses also contributed to the improvement of physicians' knowledge and practice. Physicians suggestions for improving their practice in procedural pain management included the establishment of continuous education and training programs specifically designed for health professionals involved in the care of sick neonates. The most common request was the development of national, evidence-based protocols. Moreover, many respondents highlighted the importance of parents' involvement in the care of their neonates, which is also a parental wish as demonstrated by a recent study (47).

## Limitations And Strengths

The main limitation of our study is the potential of bias associated with the social desirability and fear of identification and resultant criticism, which are inherent to surveys among professionals (48). In addition, changes in the population of physicians working in NICUs all over the country during the past 20 years might have influenced our results. Nevertheless, no significant differences were noted as regards the participants' demographics between the two time-points of the survey. The strength of our study is the monitoring of the trends as regards neonatal pain management over a period of 20 years, during which the international scientific community made a lot of effort to improve the neonatologists' understanding of neonatal pain physiology as well as the acute and long-term consequences. The high response rate ensures that our survey accurately depicts the majority of the past and current NICU-physicians' views in the country.

## Conclusions

This study showed that the beliefs of Greek physicians working in NICUs as to the neonates' capability to experience pain and stress, and the need for effective treatment of neonatal pain remained strong during a 20-year time-period. Nevertheless, despite the increased awareness of the NICU-physicians on the importance of providing adequate analgesia-sedation in critically ill infants and the overall improvement in the policies related to the prevention and management of neonatal pain over time, every day practices are still suboptimal, while important differences do exist among centers. Steps that need to be taken nationwide to further improve management of all types of pain in neonates should include continuing medical education as well as implementation of globally accepted, evidence-based protocols, integral part of which should be -apart from the pharmacological and non-pharmacological measures- the use of readily applied and reliable tools for the assessment of acute or prolonged pain and stress.

## Data Availability Statement

The raw data supporting the conclusions of this article will be made available by the authors, without undue reservation.

## Ethics Statement

Ethical review and approval was not required for the study on human participants in accordance with the local legislation and institutional requirements. The patients/participants provided their written informed consent to participate in this study.

## Author Contributions

KS and EA: conceptualized and designed the work. KT, AT, MF, and AK: data collection, analysis, and interpretation. EA, TS, and PK: drafted the manuscript. KS and EA critically reviewed the manuscript and approved the final version for submission. All authors contributed to the article and approved the submitted version.

## Conflict of Interest

The authors declare that the research was conducted in the absence of any commercial or financial relationships that could be construed as a potential conflict of interest.
