# Supplementary material for: Changes in Physicians' Perceptions and Practices on Neonatal Pain Management Over the Past 20 Years. A Survey Conducted at Two Time-Points
Source: Front Pediatr. 2021 Jun 4;9:667806. doi: 10.3389/fped.2021.667806 (PMC8211759; doi:10.3389/fped.2021.667806)
Supplement: Supplementary file 1 [file Data_Sheet_1.pdf]

SUPPLEMENTARY MATERIAL

Table 1. Study questionnaire

DEMOGRAPHIC DATA

1.Sex: male □ female □
2. Place of work: a) Public hospital □ b) Private hospital □
3. Medical speciality/subspeciality: α) Neonatologist □ β) Trainee neonatologists □ γ) Pediatrician working in a NICU □
4. Years of working in the NICU: ………………..................

Physician's perceptions

5. Do you believe that neonates are capable of feeling pain?
YES □ NO □

6. Do you believe that pain may have adverse effects in the neonate?
YES □ NO □

7. Is there a protocol for sedation and analgesia in your department?
YES □ NO □

8. Regardless of the NICU policy, you consider the administration of sedation-analgesia to neonates undergoing painful/stressful procedures as:
a) necessary □ b) important □ c) not needed □

Physician's practices

9.A. Do you use analgesia in neonates following major surgeries?
YES □ NO □
9.B. If yes, which analgesic?
a) morphine □ c) meperidine (pethidine) □
b) fentanyl □ d) paracetamol □
ε) other (define) ……………………...

10.A. Do you use sedation – analgesia to neonates receiving mechanical ventilation :
a) never □ *β)* in special occasions □ *c)* often □ *d*) always □
If the answer is the (b), please identify the special cases: ……………..……………………………………
……………………………………………………………………………………………
10.B. If you use medication during mechanical ventilation, which of the following do you use?
a) morphine □ e) diazepam □
b) fentanyl □ f) phenobarbital □
c) midazolam □ g) chloral hydrate □
d) lorazepam □ h) other (define) …..............................

11. If you use medication postoperatively or during mechanical ventilation, which way of administration do you prefer?
a) standard intervals □
b) continuous infusion □
c) on demand □

12.A. Do you administer muscle relaxants during mechanical ventilation?
a) never □ b) rarely □ c) often □ d) always □

12.B. If yes, which muscle relaxant do you use?
a) pancuronium (Pavulon) □ c) vecuronium (Norcuron) □
c) atracurium (Tracrium) □ d) other (define) ………………………

12.C. If you administer muscle relaxant to neonates on mechanical ventilation:
a) do you use a sedative and/or analgesic along with the muscle relaxant? □
b) do you believe that there is no need of sedative and/or analgesic use with muscle relaxant? □

13.A. Do you use a sedative or analgesic prior to non-emergent intubation? YES □ NO □

13.B. If yes, which of the following?
a) morphine □ f) diazepam □
b) fentanyl □ g) phenobarbital □
c) paracetamol □ h) chloral hydrate □
d) midazolam □ i) other (define) ....
e) Ketamine □

13.C. Do you use a muscle relaxant prior to non-emergent intubation? YES □ NO □

13.D. If yes, which relaxant?
a) pancuronium (Pavulon) □ c) vecuronium (Norcuron) □
b) atracurium (Tracrium) □ d) other (define) ………………………

14.A. Do you use systemic analgesia/sedation during chest drainage?.
YES □ NO □

14.B. If yes, which of the following?
a) morphine □ e) midazolam □
b) fentanyl □ f) diazepam □
c) paracetamol □ g) phenobarbital □
d) chloral hydrate □ h) sedation □
i) other (define) ....

15. Do you use systemic analgesia/sedation or local anesthesia prior to or during the following painful procedures? If yes, please define.
a) Lumbar puncture: YES □ (define …………………………………..……………..) NO □
b) Peripheral arterial line placement: YES □ (define ……………………..…………..) NO □
c) Endotracheal suction: YES □ (define ……………………………………….……..) NO □
d) Chest tube placement: YES □ (define ……………………..…………….……..) NO □
e) Suprapubic paracentesis: YES □ (define ……………………………………..……..) NO □
f) Venous puncture: YES □ (define ……………………………………..………..) NO □
g) Heel prick: YES □ (define ……………………………………………….………..) NO □
h) Other procedures (define ....………………………………......)

16.A. Do you use non-pharmacological means to alleviate pain and discomfort prior to or during minor painful or stressful procedures?
YES □ NO □

16.B. If yes, which of the following non-pharmacological means do you use?
a) minimization of procedures and handling of the neonates □
b) decrease of the light and noise in the NICU □
c) tactile stimulation □
e) skin to skin contact □
f) use of non-nutritive sucking (pacifier) □
g) sweet solutions orally □
h) other (define) ....…………………………………........

17.A. Do you use neonatal pain assessment tools? YES □ NO □

17.B. If yes, who performs the assessment? doctor □ nurse □

17.C. If yes, please indicate which of the following neonatal pain assessment scales you use:

a) NIPS (Neonatal Infant Pain Scale) □
b) PIPP (Premature Infant Pain Profile) □
c) COMFORT □
d) N-PASS (Neonatal Pain Agitation & Sedation Scale) □
e) EDIN (Echelle Douleur Inconfort Nouveau-Ne) □
f) DAN (Douleur Algus du Nouveau-Ne) □
g) CRIES (Cry, Requires oxygen, Increased vital signs, Expressions, Sleeplessness) □
h) NFCS (Neonatal Facial Coding System) □
i) other (define) ………………………………………………………………

Educational sources & respondent's suggestions

18. If your view is that neonates need sedation-analgesia, which sources of information contributed to this end.

a) International literature □ b) Greek literature □

c) Lectures at international congresses □ d) Lectures at Greek congresses □

e) Lectures in the NICU □ f) Discussions during daily ward rounds □

g) other (define)…………………………………………………

19.A. Do you think that further education of Greek neonatologists on neonatal pain management is required?

YES □, NO □

19.B. If yes, what would you suggest? ………………………………………………………………….….

…………………………………………………………………………………………….

Further comments

………
………
………
………
………
………
………

Supplementary Table 2. Independent association of the time-point with the use of analgesia – sedation and non-pharmacological measures for neonatal pain, after adjustment for physicians' sex and working years in neonatal intensive care unit.

Dependent variables	Independent variables[a]	p	Exp(B)	95% CI for Exp(B)	
				Lower	Upper
A-S post-operationally	T-P	0.137	5.431	0.584	50.536
A-S during MV	T-P	0.006	0.159	0.043	0.590
A-S for non-emergent intubation	T-P	0.053	0.511	0.258	1.010
A-S for other procedures	T-P	0.030	0.377	0.156	0.910
Local anesthetics	T-P	0.000	0.062	0.030	0.129
Non-pharmacological approaches	T-P	0.006	6.091	1.659	22.361
Sweet solutions	T-P	0.000	9.353	4.707	18.583

[a] The physicians' sex and working years in NICU were not significantly associated with any dependent variable; A-S, analgesics – sedatives; MV, mechanical ventilation; NICU, neonatal intensive care unit; T-P, time-point

Supplementary Table 3. Factors independently associated with the use of analgesia – sedation during mechanical ventilation. The time-point, physicians' sex, and working years in neonatal intensive care unit entered the model as independent variables.

Dependent variables	Independent variables[a]	p	Exp(B)	95% CI for Exp(B)	
				Lower	Upper
Opioids	T-P	0.000	5.727	2.389	13.727
	Years in NICU	0.042	1.057	1.002	1.115
Morphine	T-P	0.154	0.603	0.301	1.208
	Sex	0.031	2.231	1.075	4.632
Fentanyl	T-P	0.000	10.291	4.881	21.696
Paracetamol	T-P	0.001	2.887	1.570	5.307
General anesthetics	T-P	0.020	6.131	1.333	28.192
Sedation	T-P	0.021	2.015	1.111	3.653
Midazolam	T-P	0.000	4.570	2.368	8.821
	Years in NICU	0.019	1.048	1.008	1.089
Diazepam	T-P	0.340	0.587	0.196	1.754
	sex	0.026	3.448	1.156	10.279
Phenobarbital	T-P	0.047	8.348	1.033	67.443

[a] only T-P and significant independent variables are shown. A-S, analgesics – sedatives; NICU, neonatal intensive care unit; T-P, time-point
